# Communities without Health Departments in the Crusade against Tuberculosis

**Published:** 1904-07

**Authors:** 


### SELECTIONS AND ABSTRACTS.

### Communities Without Health Departments in the Crusade Against Tuberculosis.*

Prevention of disease as a governmental function is as yet so undeveloped as to find many governments unprepared for it. This, perhaps, is even truer of republican forms of government than of monarchical forms. Where one-man power prevails, one man alone needs to be educated for the introduction of new ideas; whereas in governments by the people the masses must be educated.

In the United States little progress has been made as yet in weaving newly discovered truths about the prevention of disease into the fabric of our laws. As a people, we still prefer to look upon disease as a matter between the individual and his God, rather than as a matter between the individual and his neighbor—as something too sacred and personal to be interfered with by any man or by any number of men. We scarcely have begun to get up machinery in any of our forms of government for the prevention of disease.

Until very recently the only kind of machinery which had been created in our governments for the prevention of disease had been what we call boards of health. These are loosely constructed committees or bodies with more or less arbitrary powers, rather legislative in character than executive, and without due responsibility to any one. They have become thorns in the flesh of the people, because they mostly have been in evidence in times of distress and have arrested public attention rather by drastic measures for stamping out epidemics than by preventing such with milder measures beforehand. Only in the United States government and a few cities of the first class has an effort been made to establish departments of health and to put prevention of disease upon a legal and scientific basis.

*Read by Lawrence F. Flick, M.D., Philadelphia, director of the Phipps Institute, before the Scranton Society for the Prevention and Cure of Consumption, on the first anniversary of its organization, February 20, 1904.

Probably all of our States, most of our cities, practically all of our towns and counties, still go lumbering along, either without any machinery for the prevention of disease or with boards of health. Such governments are seriously handicapped in the modern crusade against tuberculosis which is being preached throughout the civilized world. What can the people in communities where such governments exist do to help along the crusade?

In practice, the crusade against tuberculosis means the cure of the disease during certain stages, its prevention in all stages, and by these means its ultimate extinction. On account of the great prevalency of tuberculosis, its chronicity, and its contagiousness, the undertaking touches at many points on sociological, philanthropic, and governmental problems. It is perhaps the greatest task that has ever confronted man. As matters stand, its working out can not be the same in every place. Ways and means must vary with density of population, standard of intelligence, and grade of prosperity.

The curability of tuberculosis is well established. Skepticism upon this subject must go down before ocular demonstration. Persons cured of tuberculosis are no longer curiosities. They are to be found everywhere, and even can be seen in bunches nowadays in gatherings of many persons. Before long every community will have its fraternity of cured "lungers."

In the early stages of tuberculosis all cases recover under proper treatment. This treatment is so simple that it ought to be within the reach of every one. That it is not, is because the truth is not recognized. Men have not yet freed themselves from the shackles of the past. It is hard to unlearn. In most cases tuberculosis still remains undiscovered until death has a mortgage on the victim. The average physician still clings to cough mixtures and closed rooms, and in doing so abets the disease rather than the patient.

Even in more advanced stages, anywhere this side of consumption itself, many cases can recover. Treatment in these stages, however, is a very different proposition from treatment in the earlier stages. It implies rest, and loss of income if the patient is a working person, and it involves outlay of money for many things which need not be considered at all in earlier stage cases. The treatment, moreover, must be kept up for a very long time. When the disease has developed into consumption, treatment is a matter of prolonging life rather than of making a cure; and even that is a luxury which can be afforded only by the rich.

Tuberculosis can be cured in any climate. All that is necessary is life in the open air, proper food, well-regulated and carefully disciplined conduct, and in more advanced cases properly directed rest and exercise. People who can command these things in their homes can be cured in their homes. People who can not command them should be treated in sanatoria. Most people can be treated better in sanatoria than in their homes.

Tuberculosis is preventable as well as curable. Of this there no longer can be any doubt. It has been demonstrated that tuberculosis is due to the implantation and growth of a microorganism. The microorganism must be planted, it must grow, it must mature, and it must again be planted. Any break in this chain means a check to the disease. Stop the planting and no new cases of tuberculosis can arise.

Our knowledge of the life history of the tubercle bacillus, the microorganism mostly concerned in tuberculosis, gives us the key to the practical measures for the prevention of the disease. Tuberculosis is a house disease; it depends upon the house for implantation, growth, maturity, and seed scattering. The house is the granary of the bacillus. It is the abiding-place of the bacillus while outside of a host. It is the place where a person gets an implantation.

Sunlight, air, and water are the natural enemies of the tubercle bacillus. Sunlight and air kill it, and water dissolves it out of its *cache*, so that light and air may get at it. The bacillus comes out of a host wrapped up in broken down tissue which serves as a *cache*. When this broken down tissue is deposited in a house, it dries and hardens, and the bacilli inside remain alive for a long time. When it is deposited in the open, it is dissolved or becomes disintegrated and soon becomes devitalized. This is why an enclosure of some kind plays such an important role in the propagation of tuberculosis. In an enclosure sunlight, air, and water can not get at the tuberculous matter and it remains vital; out of doors sunlight, air, and water do get at it, and it soon becomes sterile.

Houses of one kind and another are the ordinary means of spreading tuberculosis. The home is the most frequent means and the workshop the next. This is so because it takes prolonged intimate contact with a person, place, or thing that has been intensely contaminated with tuberculous matter to give rise to an implantation. The home and the workshop are the two places where environment of sufficient intensity of contamination, and contact of long enough duration for implantation most readily can exist. Probably three-fourths of all cases of tuberculosis which are conveyed from person to person are contracted in the home, and the other fourth is contracted in the workshop.

The number of people in every community suffering from tuberculosis in various forms and stages is very large. As a rule, the poverty, helplessness, and distress of such people and of their families is great. The more densely populated the community, the larger is the number of those who suffer from the disease and the greater is the poverty and distress of the majority of those who suffer from it. To give aid to those who are struggling for life and relief to those who are suffering from the ravages of the disease is, therefore, a gigantic undertaking in any community, and the smaller the community, the more beset with difficulties it is. Provision should be made for separate treatment of early stage cases, middle stage cases, and advanced cases, and something should be done for those who are dependent upon a stricken-down breadwinner, for if not aided these will, in the near future, fill the ranks of those who are now going through the struggle.

One of the most serious difficulties in the way of providing treatment for the consumptive poor in small communities, and even in smaller cities, is the unpreparedness of the medical profession for the task. Tuberculosis has been looked upon as an incurable disease so long, that the profession as a body can scarcely be persuaded that it is curable. Very few physicians will even try to cure it. Most of them content themselves with smoothing the path of the victim of the disease in his descent to death and look somewhat askance at the man who asserts that he can cure it. So complete has been the abandonment of the victim of tuberculosis to his fate that, even at this day, medical colleges pay little attention to training men for the treatment of this disease. The result is that experts in the treatment of tuberculosis can be found only in health resorts and in large medical centres.

Experts, moreover, are not easily made. To produce an expert requires ample clinical material and long training. The hospitals connected with medical colleges do not take tuberculous subjects, and are not equipped for the treatment of tuberculosis. Ample clinical material is not available. Not until medical schools have facilities for the treatment of tuberculosis in every stage, according to modern methods, will it be possible for them to turn out medical men capable of diagnosticating tuberculosis in its early stages and treating it with prospects of recovery.

For some years to come, communities which desire to enter upon the crusade against tuberculosis will first have to train their experts. This can best be done by the establishment of dispensaries for the treatment of tuberculosis. Dispensaries, therefore, are the first essential asset in a voluntary crusade against tuberculosis. Such dispensaries will answer a threefold purpose. They will supply material to physicians for equipping themselves as experts; they will give poor people with tuberculosis, who still are able to work, an opportunity for recovery without too much sacrifice; and they will serve as a propaganda of knowledge of the prevention of tuberculosis. Such dispensaries, however, must be equipped to give material aid, as well as medical advice. They must supply medicine, preventive measure supplies, and food when necessary, and they must supervise treatment in the homes of the patients through competent visiting inspectors. They must be equipped to give baths, sterilize clothing, and do laundry work, where these are necessary for keeping the home sterile.

One of the best channels through which education can be carried on in the prevention of tuberculosis is the dispensary. It gives the entree into the houses of the poor and furnishes opportunity for instruction and control. In it many early cases which have not yet arrived at the contagious period can be cured while at work, and thus be prevented from ever becoming contagious cases. Many tuberculous subjects are compelled to work as long as they can stand up, for the simple reason that they must earn their daily bread. If such patients could only be brought under observation early enough, given a little assistance in the way of extra food, and taught the value of fresh air and cleanliness, they undoubtedly would get well. Through the inspectors of a dispensary many early cases, which otherwise would remain undiscovered, can be brought under observation. Frequently in families where only one patient in an advanced stage has applied for treatment, a number of other cases are found in various stages of the disease.

Next, communities in a volunteer campaign against tuberculosis must provide hospital accommodations for dying consumptives. A time comes in every fatal case of tuberculosis when carrying out preventive measures becomes very difficult. During the last few weeks of life a consumptive becomes quite helpless. He is no longer able to keep himself clean, and, do what he will, he is bound to soil his clothing, and especially his bed linen and body linen, with the tuberculous matter that he throws off. Usually, at this time tuberculous matter is given off in large quantities, and frequently it comes up, not only with the act of coughing, but also with that of vomiting. Under such circumstances, even a skilful nurse may find it difficult to prevent a patient from contaminating his environment. She can not prevent matter from getting on the bed linen, body linen, furniture, walls, and floor in the immediate vicinity of the bed. To make such a patient harmless, it is necessary constantly to clean that which has been soiled. This means frequent changing of bed-clothes and body linen, and scrubbing of floors and furniture. Everything must be kept spotlessly clean. In such cases it sometimes is necessary to change the linen two or three times a day. Floors have to be scrubbed daily and sometimes even mattresses have to be sterilized. Such cleanliness can not be maintained in the homes of the poor, however willing the friends and relatives may be. It is too expensive. Even charity could not afford to go into such homes and keep them sterile. The only practical way in which poor dying consumptives can be cared for is in a hospital, where the work can be done on a wholesale basis.

How small communities are to supply hospital beds for dying consumptives is a practical problem beset with many difficulties. Were it not for the prejudice which exists against consumptives and the unnatural fear which has sprung up because of the contagious nature of the disease, it is quite possible that provision might be made for most of them in existing hospitals. Our country is full of hospitals. If our hospital beds were counted and a census taken of our sick people of all kinds who can not be cared for properly in their homes, it probably would be found that we have ample beds for all. Unfortunately, however, we would rather keep our beds empty than admit cases that do not suit. Formerly, the consumptive was excluded because he lived too long and would not get well; now he is excluded because his disease is contagious. Alas that our pride should sow cockle in the wheat field of our charity! We like to give aid in distress, but we like to give it where it brings glory. Our charity has built hospitals in great numbers and has provided hospital beds which easily would give relief to all suffering humanity, but we desire to see the people whom we put into these beds get well quickly. For the short sufferer we have a welcome, but against the long sufferer we turn our faces.

In commnnities where hospitals exist an effort should be made to equip all vacant beds for dying consumptives in separate wards. This can be done without in the least degree prejudicing the interests of other patients and without endangering any one by reason of contagion. A change of this kind does not involve a great outlay of money. It would not materially add to the expense of administration. The charitable instincts of the people which such action would touch would supply the money necessary for maintaining the beds.

In communities where there are no general hospitals, or where existing hospitals refuse to make provision for dying consumptives, effort should be made to get up hospitals for the specific purpose of taking care of such cases. Hospital buildings for this purpose need not be elaborate or ornamental. The simplest construction may be used with plain equipment. All that is necessary is to house those people comfortably under proper supervision and cleanliness. The building must be so constructed that it can easily be kept clean and well ventilated. Everything in it must be plain and easily cleaned.

As a matter of economy and efficient administration, hospitals for advanced cases of tuberculosis should be run in conjunction with dispensaries for walking cases. When the two are combined the medical and nursing staff of the one can be used for the other. This arrangement is of great value in the control of patients in their homes. The doctors and nurses, by reason of their training and experience in the hospital, will be better able to supervise and control patients in their homes, and thus to enforce the carrying out of preventive measures. The nurses, moreover, will likewise profit by the arrangement, as they will be given outdoor employment part of the time of their service, and, by seeing the environments of these poor people in their homes, will be broadened in their sympathies.

The next step of a community in a voluntary campaign is to establish sanatoria for curable cases of tuberculosis which need either comparative or absolute rest. Among the poor a tuberculous subject becomes an impediment to the healthfulness and prosperity of his family the moment he is unable to work. In such families every one contributes his share to the common fund which maintains the family. When one drops out of the earning squad, the earnings of the others must be spread out to help to maintain the one who has dropped out. If this one needs rest, it means taking another out of the earning squad to wait on him. The result is that the stricken one is not looked after in a way that will give results until he is laid low by the disease, and then it is too late for recovery. Hence the vast majority of curable cases of tuberculosis among the poor are curable only in a sanatorium. Conditions for cure are unattainable in the home of the patient.

The treatment of tuberculosis, moreover, is an expensive matter, whether at home or in a sanatorium. It is beyond the reach of the poor. Good food and a comfortable, clean, well-ventilated place of abode are necessary for it. The food alone costs about four dollars a week. Care of the place of abode—preventing it from getting comtaminated and keeping it clean—even when done on a wholesale basis, costs about a dollar a week. Add to these items, medical attendance, medicine, and nursing, and we get a footing up not far short of ten dollars a week. This expense, moreover, must be borne week in and week out for from six months to two or three years.

When practicable, separate sanatoria should be established for early stage cases and for more advanced cases. Not only would the results be better under such a system but administration would be cheaper. In early stage cases comparative rest only is necessary. All early stage cases can do some work and gradually acquire a capacity for doing a great deal of work. They need no nursing and comparatively little medical attendance. Under good discipline and management they all can do their own work, and therefore need no servants. If the sanatorium is properly located, they even can contribute something toward their own support by light farming. As all are of a class and progress rapidly towards recovery they exercise a stimulating influence upon each other. With more advanced cases mixed in with other cases, on the other hand, the entire institution has to be run on a basis suitable to the more advanced cases. Nursing and close medical attendance are necessary, and there must be a full equipment of domestics. Those who can work will not want to work, because of the example of those who can not work. Stationary and retrogressive cases will have a depressing influence on all others.

It is true, many communities are too small to have separate sanatoria for different stage cases, but under such circumstances it would be well for neighboring communities to join forces. Inhabitants in two, three, or four adjoining counties could unite in the support and management of such institutions, and locate them where they would be most accessible to the majority and could be most inexpensively administered. These sanatoria could be located anywhere on dry, porous soil irrespective of elevation or climate. Perhaps the best way of uniting the inhabitants of such counties would be by organizing them into societies for this purpose. These societies, moreover, could be used for educational work, such as distributing tracts and giving lectures on the prevention of tuberculosis.

Contaminated houses are a prolific means of spreading tuberculosis. A house which has been inhabited by a consumptive who has disregarded preventive measures may give the disease to subsequent occupant. An earnest effort should, therefore, be made to prevent contamination of houses, by teaching all consumptives proper preventive measures and showing them how to carry these measures into effect. This can be accomplished in part, at least, through competent inspectors, especially when aid is given, such as food, and preventive measure supplies for convenient and easy practice of prevention. The simpler and easier the prevention of tuberculosis is made for the poor and ignorant, the more likely it is to be carried out.

Where the prevention of contamination of houses can not be carried out, something must be done toward the sterilization of houses which have been contaminated. Sterilization after contamination is very simple, but somewhat expensive. Often it implies thorough cleansing and renovation. Floors and woodwork have to be scrubbed, walls have to be scraped and repapered or repainted, and everything in the house which has been exposed to contamination has to be thoroughly cleansed. When everything has been well cleansed, the rooms have be sprinkled with formalin or filled with formaldehyde gas. All this is troublesome and costly, and because it is so it will not be done except under pressure either moral or legal. Where there is no health department legal pressure can not be brought to bear, but moral pressure may be brought to bear in many instances. When the occupant of the house is the owner a knowledge of the danger from contamination to the other members of his family will be sufficient incentive to make him do what is necessary. When the occupant is merely a tenant, public sentiment may induce the landlord to do what is necessary, if not from a sense of right, at least from a sense of commercial interest. As soon as the public shall have become sufficiently enlightened so that people will refuse to occupy houses that have been occupied by consumptives until the landlord can show a clean bill of health, all contaminated houses will be sterilized.

In communities without departments of health it is somewhat difficult to get at the location of consumptives, and the prevention of contamination of houses, as well as the sterilization of houses which have been contaminated, is for this reason surrounded with unusual difficulties. These difficulties are not insurmountable, however. It is true, compulsory registration of tuberculosis can not be resorted to, for this is a governmental function, which can only be exercised by a health department. Much can be done, however, in the way of bringing to light, through the instrumentality of dispensaries, hospitals and sanatoria, people suffering from tuberculosis. Such institutions will attract the consumptive poor and will make it possible to get almost a complete census of the consumptives of a community, in the course of time. Through those who come to such institutions others can be reached, and if a community is well organized, in due time every consumptive can be brought under observation and at least be taught what to do to prevent the spread of disease.

A society organized for the various purposes named could also accomplish something toward the prevention of tuberculosis by interesting itself in the sanitary improvement of dwelling-houses and workshops: Homes should be made clean, dry, airy and bright, both among the rich and the poor; and where the homes of the poor do not possess these qualities they should be condemned by public sentiment and prevented from being used any longer. Buildings in which men and women are employed should be made sanitary and brought up to a correct standard of air supply and light supply. Along all of these lines much can be accomplished in any community by the creation of a public sentiment.

A pertinent question that will be asked at once by all people of a practical turn of mind in connection with the scheme which I have outlined will be, Where is the money to come from to do all this with? There are in every community men and women who are willing to do a great deal for charity and philanthropy, *provided it is clear to them that the money which they give is judiciously expended and yields fruit in practical results. If the right effort is made, the money which is necessary can be raised.* Inasmuch as tuberculosis is a disease which comes home to every one, at one time or another somewhere in his family or among his friends, resources for a campaign of this kind can be drawn from practically every member of a community. Wherever an effort has been made to raise money for such a purpose it has been successful. With earnest endeavor at least enough can be raised to make a beginning, and usually such undertakings grow as people see the good that is accomplished by them.

In conclusion it may not be amiss to call attention to a method of raising money for purposes of this kind which has been tried successfully in another country—namely, Canada—and which might perhaps, easily be grafted upon our American States. This is the enactment of a State law under which any town, county, or city may get a certain amount of State aid for work of this kind, in proportion to the amount of work which the community itself does. Under such a law, if a community would establish an institution for the treatment of tuberculosis, it could draw from the State a certain amount of money in proportion to the amount of money raised for the purpose by the community; and for every patient treated it would get from the State a certain percentage of maintenance money for each day during which such patient was treated. A State law of that kind in Pennsylvania would enable many of the smaller cities, towns, and country districts to get up institutions for the treatment and prevention of tuberculosis which without such aid, will have not the courage to begin.

